# Loss of the Habenula Intrinsic Neuromodulator Kisspeptin1 Affects Learning in Larval Zebrafish

**DOI:** 10.1523/ENEURO.0326-16.2017

**Published:** 2017-05-19

**Authors:** Charlotte Lupton, Mohini Sengupta, Ruey-Kuang Cheng, Joanne Chia, Vatsala Thirumalai, Suresh Jesuthasan

**Affiliations:** 1 Department of Animal and Plant Sciences, University of Sheffield, Sheffield, S10 2TN, UK; 2 Institute for Molecular and Cell Biology, 138673, Singapore; 3 National Centre for Biological Sciences, Tata Institute of Fundamental Research, Bangalore, 560065, India; 4 Lee Kong Chian School of Medicine, Nanyang Technological University, 636921, Singapore; 5 National University of Singapore Graduate School for Integrative Sciences and Engineering, 117456, Singapore; 6 Duke-NUS Graduate Medical School, 169857 Singapore

**Keywords:** Calcium imaging, electrophysiology, habenula, intrinsic neuromodulation, mutant, operant learning

## Abstract

Learning how to actively avoid a predictable threat involves two steps: recognizing the cue that predicts upcoming punishment and learning a behavioral response that will lead to avoidance. In zebrafish, ventral habenula (vHb) neurons have been proposed to participate in both steps by encoding the expected aversiveness of a stimulus. vHb neurons increase their firing rate as expectation of punishment grows but reduce their activity as avoidance learning occurs. This leads to changes in the activity of raphe neurons, which are downstream of the vHb, during learning. How vHb activity is regulated is not known. Here, we ask whether the neuromodulator Kisspeptin1, which is expressed in the ventral habenula together with its receptor, could be involved. *Kiss1* mutants were generated with CRISPR/Cas9 using guide RNAs targeted to the signal sequence. Mutants, which have a stop codon upstream of the active Kisspeptin1 peptide, have a deficiency in learning to avoid a shock that is predicted by light. Electrophysiology indicates that Kisspeptin1 has a concentration-dependent effect on vHb neurons: depolarizing at low concentrations and hyperpolarizing at high concentrations. Two-photon calcium imaging shows that mutants have reduced raphe response to shock. These data are consistent with the hypothesis that Kisspeptin1 modulates habenula neurons as the fish learns to cope with a threat. Learning a behavioral strategy to overcome a stressor may thus be accompanied by physiological change in the habenula, mediated by intrinsic neuromodulation.

## Significance Statement

Learning to deal with adversity can positively affect one’s ability to cope with challenges in the immediate future. Control thus causes short-term change in the brain. Here, we show that the neuromodulator Kisspeptin1 is involved in learning to avoid a punishment. The expression pattern of this gene and electrophysiological recordings suggest that this molecule may function by modulating the ventral habenula, a region of the brain that mediates fear by regulating serotonin release. Kisspeptin1 could thus be a potential player in resilience developed as a result of control, extending previous findings that it can reduce fear.

## Introduction

When faced with an aversive stimulus, animals respond in a manner that is dependent on context and experience. On first encountering a threat in a novel environment, there may be panic and poorly directed attempts at escape. If the animal repeatedly encounters the threat and becomes familiar with a safe escape route, it will be able to quickly remove itself from danger. Better still, if the animal is able to recognize a cue that reliably predicts the impending threat, it will be able to escape before the aversive stimulus is present. This, in essence, is the phenomenon of active avoidance. Several theories have been proposed to explain the mechanism underlying active avoidance. In the two-factor theory, the animal first develops a fear of the conditioning stimulus (CS) that is paired with the aversive stimulus, by Pavlovian conditioning. Cotermination of the CS and the threat [unconditioned stimulus (US)] then drives learning. Expectation has a critical role, and actions that lead to better-than-predicted outcomes are reinforced (Maia, 2012). The first time an aversive stimulus is encountered, the predicted outcome would be negative. However, if an escape route has been learned, then the predicted outcome becomes positive.

How is active avoidance implemented in the brain? One structure that may be involved is the lateral habenula. As first shown in monkeys, unexpected punishment leads to increased activity in lateral habenula neurons, whereas an unexpected reward leads to inhibition (Matsumoto and Hikosaka, 2007, 2009). Direct evidence for an involvement of this structure in active avoidance comes from zebrafish, in which the homolog of the lateral habenula is termed the ventral habenula (vHb; Amo et al., 2010). Silencing vHb neurons reduces the ability of zebrafish to learn active avoidance (Amo et al., 2014). In the naive animal, unexpected punishment leads to phasic activity in vHb neurons (Amo et al., 2014). As the animal learns to associate a CS with the threat, there is tonic firing in response to the CS in vHb neurons. This causes excitation of serotonergic neurons in the dorsal raphe. As the animal learns to escape, there is decreased tonic firing. Tonic activity in the vHb has thus been proposed to encode aversive reward expectation value, and learning active avoidance is associated with a change in vHb activity in response to the CS (Amo et al., 2014).

What is the mechanism of change in vHb activity as learning occurs? Amo et al. (2014) have proposed that excitation is regulated via feedback from serotonergic neurons in the raphe to the entopeduncular nucleus, which is the teleost homolog of the basal ganglia (Turner et al., 2016). Whether additional mechanisms are involved is unknown. Here, we examine the possibility that a change in habenula neurons accompanies the learning process. In particular, we examine the potential involvement of the neuromodulator Kisspeptin1, which is expressed in the vHb together with its receptor (Kitahashi et al., 2009). In zebrafish, two paralogs of *kiss1* have been identified. These have nonoverlapping expression, with *kiss1* being restricted to the habenula and *kiss2* expressed in the hypothalamus and posterior tuberculum (Kitahashi et al., 2009). This allows a specific test of the role of *kiss1* in the habenula using genetics.

In mammals, *kiss1* is expressed in the hypothalamus and is well studied in the context of reproduction (Pinilla et al., 2012; Clarke et al., 2015). Burst firing of hypothalamic neurons leads to the release of Kisspeptin1 (Kelly et al., 2013), which causes sustained depolarization of gonadotropin-releasing hormone (GnRH) neurons (Han et al., 2005). Kisspeptin1 is also expressed in the hippocampus, where it causes an increase in EPSC amplitude and contributes to increased excitability (Arai, 2009). In the zebrafish habenula, Kisspeptin1 has been proposed to depolarize vHb neurons, based on its ability to induce *c-fos* expression (Ogawa et al., 2014). However, delivery of Kisspeptin1 decreases fear, which is inconsistent with evidence that excitation of vHb neurons is aversive (Amo et al., 2014). Moreover, killing Kisspeptin1 receptor–expressing neurons in ventral habenula neurons mimics the effect of Kisspeptin1 delivery (Ogawa et al., 2014), which would not be expected if Kisspeptin1 causes depolarization. Rather, these observations suggest that Kisspeptin1 can also cause hyperpolarization. Here, we ask whether Kisspeptin1 could be involved in learning avoidance, by assessing the ability of a *kiss1* mutant to learn and whether Kiss1 has the ability to both depolarize and hyperpolarize vHb neurons, which would make Kiss1 signaling a potential mechanism for alteration of vHb neuron activity during avoidance learning.

## Materials and Methods

### Animals

Experiments were conducted on the *nacre* strain of zebrafish, *Danio rerio*, in accordance with protocols approved by the Institutional Animal Care and Use Committee. Animals at the stages tested could not be sexed.

### Antibody labeling

Standard methods were used. Briefly, fish were fixed overnight at 4°C in 4% paraformaldehyde in PBS. A solution of PBS with 1% bovine serum albumin (Fraction V; Sigma-Aldrich), 1% DMSO, and 0.1% Triton X-100 was used to permeabilize the tissue and dilute primary antibodies. The antibodies to Kisspeptin1 (Parhar Lab, Monash University Malaysia, PAS 15133/15134; RRID:AB_2490069) and the receptor Kissr1b have been described previously (Servili et al., 2011; Nathan et al., 2015a). Alexa Fluor 488–conjugated goat anti-rabbit antibodies (Thermo Fisher Scientific, A11078, lot 792513; RRID:AB_10584486) were used at 1:1000 dilution in PBS. Imaging was conducted using a Zeiss LSM510 confocal microscope with a 40× water immersion objective.

### Mutagenesis of the *kiss1* locus

Guide RNAs to the *kiss1* gene were designed using ZiFiT Targeter (Sander et al., 2007). The Basic Local Alignment Search Tool (BLAST) was used to test for off-targets, and only those target sites that yielded no identical off-targets were used. The chosen target sites were integrated into a forward primer (GAAATTAATACGACTCACTATAGGN_18_GTTTTAGAGCTAGAAATAGC; Bassett et al., 2013). PCR was performed with Phusion High-Fidelity polymerase (Thermo Fisher Scientific) and a universal reverse primer that defined the remainder of the single-guide RNA (sgRNA) sequence (AAAAGCACCGACTCGGTGCCACTTTTTCAAGTTGATAACGGACTAGCCTTATTTTAACTTGCTATTTCTAGCTCTAAC). PCR products were purified (QIAquick PCR purification kit; Qiagen) and 0.1 μg was transcribed using the MEGAshortscript T7 Transcription Kit (Invitrogen). sgRNAs were purified using ammonium acetate precipitation [2.5 µL of 0.1 m EDTA (Promega), 5 µL of 5 m ammonium acetate solution (Qiagen), 115 µL of 100% ethanol)] and stored in 1-µL aliquots at –80°C.

The CRISPR/Cas9 expression vector pT3Ts-nls-zCas9-nls (Addgene plasmid #46757; Jao et al., 2013) was linearized using XBaI (New England Biolabs). Cas9 mRNA was produced by *in vitro* transcription of 1 µg template using the mMessage mMachine T3/T7 kit. Capped, polyadenylated Cas9 mRNA was made using the Poly(A) kit (Ambion). The reaction was precipitated using 30 µL lithium chloride solution (Ambion). RNA was eluted in 30 µL nuclease-free H_2_O, aliquoted, and stored at –80°C until use. A 1-µL sample was run on a 1% agarose gel alongside the RiboRuler High Range ladder (Thermo Fisher Scientific) to check for correct size of product (∼3 kb).

A mixture containing ∼1 µg Cas9 mRNA and 400 ng/µL of sgRNAs was injected into the animal pole of embryos from a *elavl3:GCaMP6f* transgenic line (Cheng et al., 2016) at the one-cell stage. To assess the effects, high-resolution melt analysis (HRMA) was performed using the MeltDoctor HRM Mastermix [Invitrogen, 5 µL MeltDoctor HRM Mastermix, 1 µL diluted DNA (20 ng/µL; obtained by digesting 24-h embryos in 20 µg/µL proteinase K at 55°C for 60 min), 0.3 µL forward and reverse primer (10 µM), and 3.4 µL nuclease-free H_2_O] under the following conditions: 95°C 10 min, 40 cycles of [95°C 15 s, 60°C 1 min], 95°C 10 s, 60°C 1 min, 95°C 15 s, 60°C 15 s on the 7500 Fast Real-Time PCR system (Applied Biosystems). Nucleic acid concentration was measured using a Nanodrop ND-1000 (Thermo Fisher Scientific). Concentrations of nucleic acids were measured by the absorbance at 260 nm, and the purity of the sample was quantified by the ratio of sample absorbance at 260/280 nm. A lower limit of 1.10 was set as the boundary for an acceptable reading for the 260/280 values because of impurities interfering with the HRMA analysis. During each HRMA reaction, three wild-type samples were used as controls, and each test sample was run as a replicate.

### Active avoidance conditioning

Conditioning was conducted using a two-way chamber, as described in Cheng and Jesuthasan (2012), with the addition of a separator made of matte black cardboard between the two compartments. Before the start of conditioning, fish were allowed to habituate in the chamber for 20 min. The CS, a red LED, was delivered for 8 s, and the US, a 25-V pulse, was delivered for 100 ms using a Grass SD9 stimulator. An interval of at least 6 min was provided between trials; the next trial commenced only when the fish entered the target area, which is the quadrant near the side wall. The entire training session normally lasted 90 min. Fish were aged 4–6 weeks, as younger fish could not learn this type of behavior (Valente et al., 2012). Fish were genotyped after the assay by sequencing. Performance in the assay was determined by a computer (i.e., blind to genotype).

### Statistical analysis

Data structure was assessed using the Shapiro–Wilks test, and effect size was measured using Cohen’s *d* for normally distributed data or with *r* for non–normally distributed data. To assess *p* values, Student’s *t test* was used for data with a normal distribution; otherwise, Mann–Whitney *U* test or Wilcoxon matched-pairs test was applied.

### Electrophysiology

Whole-cell patch-clamp recordings were performed from vHb neurons in 6–8 days postfertilization (dpf) larvae using procedures described in Sengupta and Thirumalai (2015). Briefly, larvae were anesthetized in 0.01% MS222 and pinned onto a Sylgard (Dow Corning) dish using fine tungsten wire (California Fine Wire). The MS222 was then replaced with external saline (composition in mm: 134 NaCl, 2.9 KCl, 1.2 MgCl_2_, 10 Hepes, 10 Glucose, 2.1 CaCl_2_, 0.01 d-tubocurare, pH 7.8, 290 mOsm), and the skin over the head was carefully peeled off to expose the brain. The recording chamber was then transferred to the rig apparatus. All recordings were done in an awake, *in vivo* condition. The cells were observed using a 60× water immersion objective on a compound microscope (Olympus BX61WI). Cells in the ventral most layer of the habenula and on the lateral most side were targeted for recordings, as these cells express the Kiss1 receptor. The ventral neuropil of the habenula was used as a landmark. Pipettes of tip diameter 1–1.5 μm and resistance of 12–16 MΩ were pulled with thick-walled borosilicate capillaries (1.5 mm OD; 0.86 mm ID; Warner Instruments) using a Flaming Brown P-97 pipette puller (Sutter Instruments). A potassium gluconate–based patch internal solution (composition in mm: 115 K gluconate, 15 KCl, 2 MgCl_2_, 10 Hepes, 10 EGTA, 4 MgATP, pH 7.2, 290 mOsm) was used for all recordings. Whole-cell recordings were acquired using Multiclamp 700B amplifier, Digidata 1440A digitizer, and pCLAMP software (Molecular Devices). The data were low-pass filtered at 2 kHz using a Bessel filter and sampled at 20 kHz at a gain of 1. Membrane potentials mentioned were not corrected for liquid junction potential, which was measured to be +8 mV for the potassium gluconate–based internal solution. The following substances were perfused in the bath: tetrodotoxin (1 μm, Alomone labs, Israel), zebrafish K10 (Isca Biochemicals), and K234 (Isca biochemicals). Events were detected offline using Clampfit (Molecular Devices). Graphs were plotted using Microsoft Excel. Statistical analysis was performed using Matlab.

### Calcium imaging

Zebrafish larvae (6–8 dpf) expressing GCaMP6f under the *elavl3* promoter were anesthetized in mivacurium and embedded in low-melting-temperature agarose (2% in E3: 5 mm NaCl, 0.17 mm KCl, 0.33 mm CaCl_2_, 0.33 mm MgSO_4_) in a glass-bottom dish (Mat Tek). They were imaged at 1 Hz on a Nikon two-photon microscope (A1RMP) on a fixed stage upright microscope using a 25× water immersion objective (NA = 1.1). The femtosecond laser (Coherent Vision II) was tuned to 920 nm. The stimulus was delivered using a Grass SD9 stimulator. Fish used in this experiment were derived from offspring of kiss1^sq1sj^ heterozygotes and were genotyped by PCR after imaging.

### Analysis of calcium imaging data

Cells in the superior raphe were outlined manually in ImageJ to create regions of interest (ROIs). The ratio f/f_0_ for all ROIs was obtained, where f_0_ is the average intensity in a 15-s period before stimulus onset. The average ratio for each cell in the 15-s period after stimulus onset was calculated. Estimate of the intracluster correlation and multilevel analysis was conducted as described in Aarts et al., (2014), using R software.

## Results

**Figure 1.**
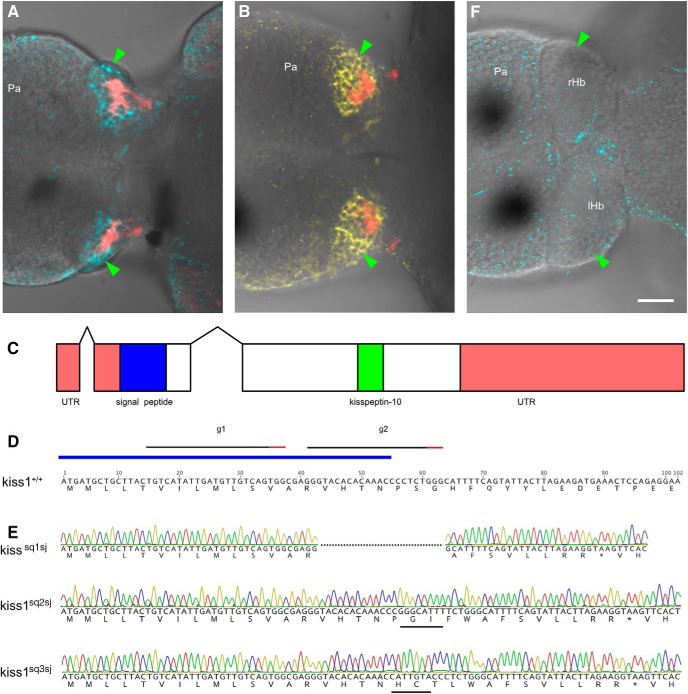
Generation of mutations in the zebrafish *kiss1* gene using CRISPR/Cas9. ***A***, ***B***, Dorsal view of zebrafish larvae, labeled with an antibody to Kisspeptin1 (***A***; cyan) and the Kisspeptin receptor (***B***; yellow). These fish are from the *Et(SqKR11)* transgenic line (Lee et al., 2010) and express red fluorescence in afferents from the entopeduncular nucleus (basal ganglia) that terminate in the neuropil of the ventral habenula. The green arrowhead indicates the habenula. The speckles appear to be nonspecific label. ***C***, A schematic of the *kiss1* gene. There are two introns. The signal sequence is shown in blue, and the region containing the active Kisspeptin1 peptide is shown in green. ***D***, Partial sequence of the *kiss1* gene. The signal sequence is indicated by the blue bar. The position of the two guide RNAs are indicated by the black bars. ***E***, The sequence of the three mutant alleles, together with predicted translations. The asterisks indicate stop codons. The black bars indicate inserted sequences. ***F***, A *kiss1*^sq1sj−/−^ fish, following labeling with the Kisspeptin1 antibody. No signal could be detected in the habenula. Scale bar = 25 µm. Pa, pallium; rHb, right habenula; lHb, left habenula. Images are single optical sections, with anterior to the left.

As in adult zebrafish (Servili et al., 2011; Nathan et al., 2015a), Kisspeptin1 is present in the ventral habenula of larval zebrafish, together with its high-affinity receptor Kiss1ra (Onuma and Duan, 2012; Fig. 1*A*, *B*). The Kisspeptin system is thus expressed in an appropriate manner to locally regulate vHb neurons even at an early developmental stage. If Kisspeptin1 signaling is involved in active avoidance learning, fish that lack Kiss1 should be deficient in this process. To test this, we generated mutations in the *kiss1* locus using CRISPR/Cas9 (Hruscha et al., 2013; Hwang et al., 2013). Two guide RNAs were designed to the signal peptide region of Kisspeptin1 (Fig. 1*C*). These were injected into embryos at the one-cell stage, together with mRNA for Cas9. HRMA of genomic DNA derived from injected embryos indicated that the guide RNAs were effective. This was confirmed by sequencing: eight of eight injected embryos contained mutations at the target site. Injected siblings were grown to adulthood, and sequencing of F1 fish indicated a transmission rate of 100%. Three alleles were obtained: one consisted of a 20-bp deletion (*kiss1* ^sq1sj^; Fig. 1*D*, *E*), whereas the other two were insertions of 7 and 8 bp (*kiss1*^sq2sj^ and *kiss1*^sq3sj^); *kiss1*^sq3sj^ also contained a 1-bp deletion, resulting in the same reading frame as *kiss1*^sq2sj^. All alleles gave rise to premature stop codons upstream to the Kisspeptin1 peptide (Fig. 1*E*). No label could be detected in mutant fish by immunofluorescence with an antibody to the C terminus of prepro-Kisspeptin1 (Servili et al., 2011; Fig. 1*F*), further indicating that the mutants lacked the peptide.

**Figure 2.**
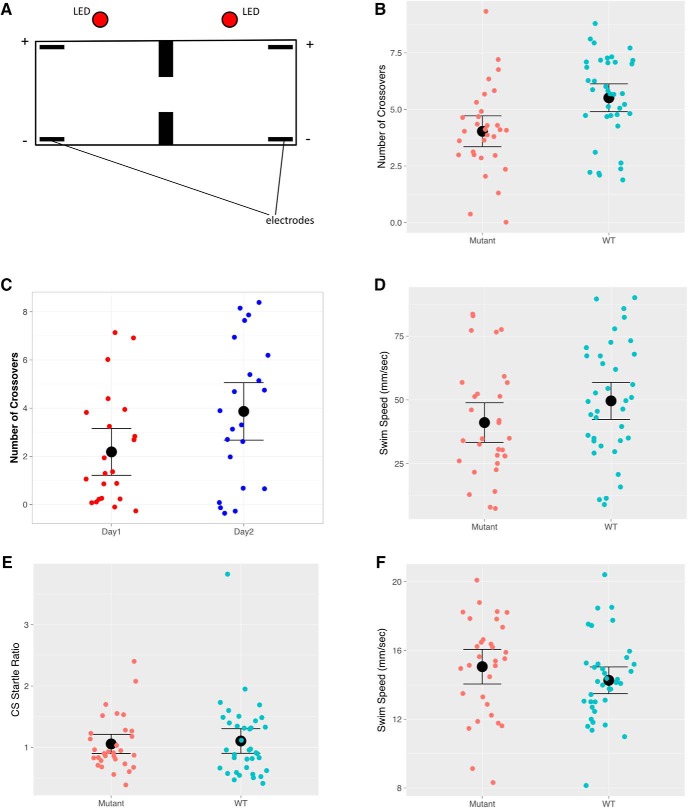
The behavior of *kiss1* mutants in an active avoidance assay. ***A***, Schematic of the two-way active avoidance chamber. ***B***, Number of crossovers before shock delivery for mutants (*n* = 31) and WT siblings (*n* = 37). The black symbols are average values. ***C***, Number of crossovers in a group of mutants (*n* = 22) trained over two consecutive days. ***D***, Response to the first shock, as measured by swim speed in the first second immediately after the shock. ***E***, Mean swim speed during the 20-min habituation period. ***F***, Response to the first exposure to the red light. This was measured by the ratio of the mean speed in the 2 s after light onset to the mean speed in the 5 s before light onset. In all cases error bars indicate 95% confidence interval.

To test their ability to learn to avoid an aversive stimulus, juvenile mutants and wild-type animals (4–6 wks of age) were tested individually in a tank with two compartments (Fig. 2*A*), similar to a previously described apparatus (Lee et al., 2010; Cheng and Jesuthasan, 2012), but with the addition of a partial separator between the compartments. Each compartment contained a red light (the CS) and electrodes that can deliver an aversive shock. The light was turned on for 8 s in the compartment containing the fish and coterminated with the shock if the fish remained in the CS side at the end of the CS presentation. If the fish moved to the non-CS side and stayed there until the end of the CS presentation, no shock was delivered. Fish were exposed to 10 training trials. The cross score was defined as the number of trials in which an individual fish swam to the non-CS chamber before the light was turned off. *Kiss1* mutants showed a lower cross score compared with wild types (Fig. 2*B*; *p* = 0.0024, *d* = 0.77; Table 1). We also trained a separate group of mutants in the same task over two consecutive days. As shown in Fig. 2*C*, a significantly better performance was observed on the second day of training compared with the performance on the first day (*p* = 0.011, *d* = 0.91). These observations suggest that loss of Kiss1 does not abolish the fish’s learning ability but contributes to suboptimal performance in learning active avoidance.

**Table 1.** Statistical analysis performed

Figure	Data structure	Type of test	Result
2*B*	Normal distribution	Shapiro–Wilk test	*W* = 0.969; *p* = 0.0929
		*t* test	*p* = 0.0024
		Effect size (Cohen’s *d*)	*d* = 0.77
2*C*	Nonnormal distribution	Shapiro–Wilk test	*W* = 0.889; *p* = 0.001
		Wilcoxon matched pairs test	*p* = 0.011
		Effect size (adjusted Cohen’s *d*)	*d* = 0.91
2*D*	Normal distribution	Shapiro–Wilk test	*W* = 0.966; *p* = 0.057
		*t* test	*p* = 0.13
2*E*	Normal distribution	Shapiro–Wilk test	*W* = 0.986; *p* = 0.673
		*t* test	*p* = 0.22
2*F*	Nonnormal distribution	Shapiro–Wilk test	*W* = 0.829; *p* = 0.001
		Mann–Whitney *U* test	*p* = 0.98
3*C*	Nonnormal distribution	Kruskal–Wallis nonparametric test followed by *post hoc* Tukey–Kramer method of multiple comparison	*p* = 0.012; *p* = 0.029 (10 nm vs. 5 µm); *p* = 0.034 (100 nm vs. 5 µm)
4*B*	Multilevel model analysis	Intracluster correlation	0.46
		Chi-squared (df = 1)	5.5971
			*p* = 0.015

Kiss1 has been shown to reduce the behavioral response to an aversive stimulus (Ogawa et al., 2014). Thus, mutants may have increased behavioral response to the shock. To test this, we analyzed the swim speed immediately after the onset of the first electrical shock. As shown in Fig. 2*D*, both mutants and wild type responded to the shock with an increase in swim speed (compare with the swim speed before the shock: Fig. 2*F*). There was no significant difference between the two groups (*p* = 0.13). We also asked whether mutant fish have increased anxiety. Anxious fish generally behave differently in a novel environment. We thus compared the swim speed in the 20-min habituation period immediately after fish were introduced to the test tank. As shown in Fig. 2*E*, no difference was observed between mutant and wild type (*p* = 0.22). We also assessed the response of the fish to the red LED, as anxious fish may respond to this with a startle (Lee et al., 2010). No difference was seen (Fig. 2*F*, *p* = 0.98). Collectively, the data suggest that there was no behavioral difference in the response to the shock, the red light, or the novel environment in the beginning of the task.

**Figure 3.**
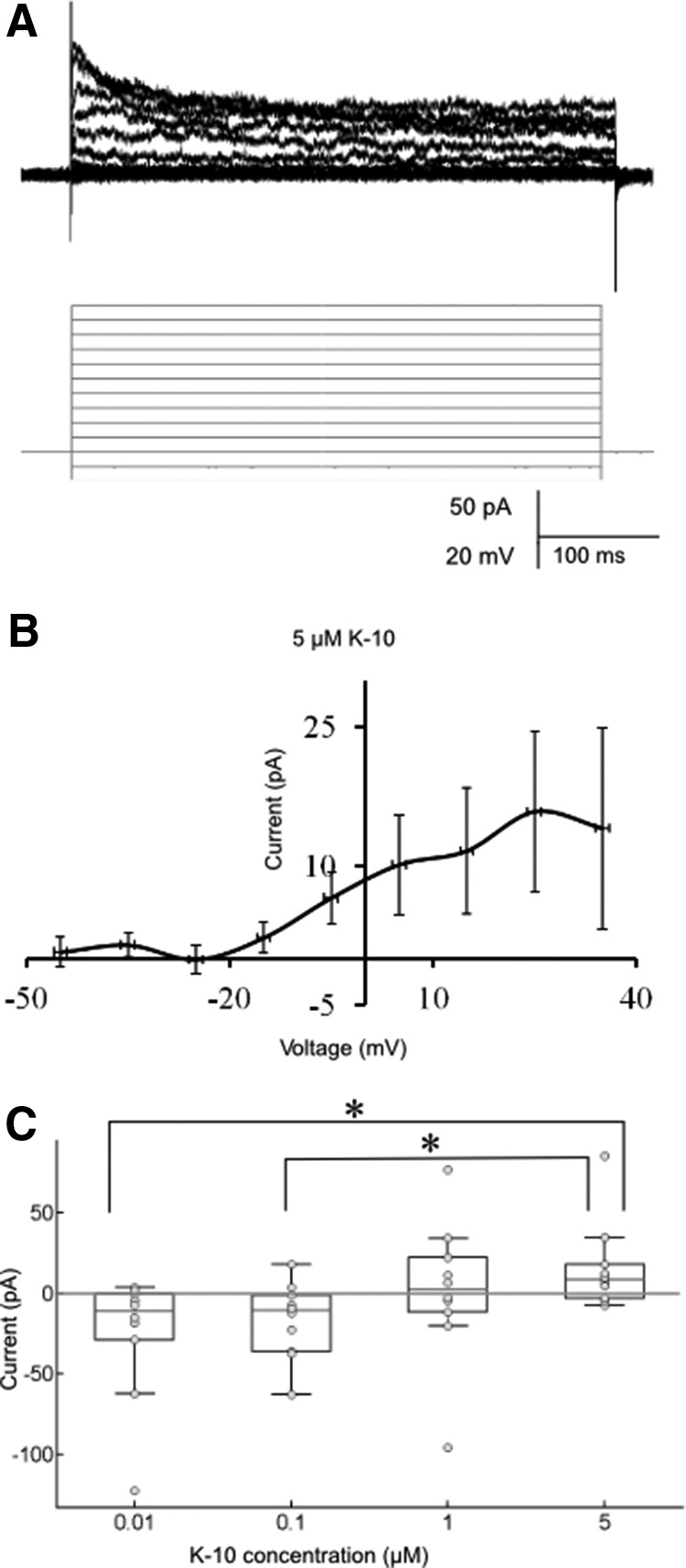
The effect of Kisspeptin1 on vHb neurons. ***A***, Representative trace of cellular response (top, black) in a cell to voltage steps (bottom, gray). These recordings were done in the presence of 1 μm tetrodotoxin to block network activity. ***B***, Difference current obtained after bath application of 5 μm K-10 (*n* = 10 cells). The same protocol as in ***A*** was done before and after bath application of K-10 for each cell. The traces obtained after were subtracted from the traces obtained before application of the peptide for the same cell. ***C***, Dosage response of the difference current. Superimposed scatter and box plots for the difference currents induced at 25 mV for four different K-10 concentrations (10 nm, 100 nm, 1 μm, and 5 μm). Difference currents induced at 10 and 100 nm are significantly different from that induced at 5 μm (*p < 0.05, Kruskal–Wallis nonparametric test followed by *post hoc* Tukey–Kramer method of multiple comparison, *n* = 10 cells for each K10 dose).

To determine how Kisspeptin1 affects habenula neurons, we performed whole-cell patch-clamp recordings from these cells in 6- to 8-dpf wild-type larvae. We bath-applied zebrafish K-10, a 10–amino acid active peptide of Kisspeptin1. 1 μm tetrodotoxin was added to the bath solution to globally block network activity. Cells were recorded in voltage clamp mode and were taken through a series of 500-ms voltage steps (Fig. 3*A*, lower panel). The cellular response (Fig. 3*A*, upper panel) was recorded before and after bath application of K-10. The recording was allowed to stabilize for 5 min in normal saline before K-10 was applied. Input resistances and holding currents did not change significantly with application of K-10 (*p* = 0.486 for holding currents and *p* = 0.587 for input resistances, sign test, *n* = 40 cells). Difference currents were then calculated by subtracting the current values after K-10 application from the one before K-10 application (control). This indicates that 5 μm K-10 induced an outward current at depolarized potentials (Fig. 3*B*, *n* = 10 cells from 7-dpf larvae). The mean peak amplitude of this current at 25 mV was 15.8 ± 8.7 pA. To determine whether the Kisspeptin1-evoked current was dose dependent, we repeated the experiment with 10 nm, 100 nm, and 1 μm K10. Lower concentrations of K-10 application induced lower amplitudes of the peak current at 25 mV (Fig. 3*C*), with 100 nm inducing an inward current, suggesting that low and high concentrations of Kisspeptin1 have opposite effects on habenula neurons. This suggests that Kisspeptin1 has concentration-dependent effects on vHb neurons.

**Figure 4.**
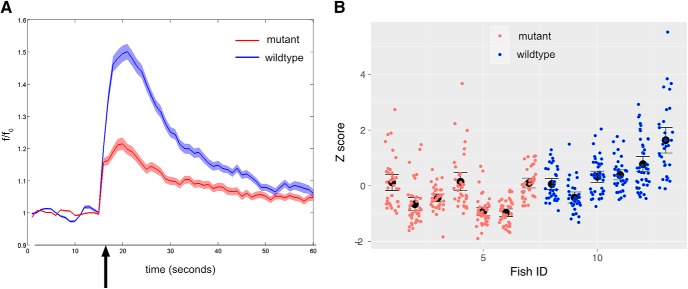
The effect of shock on raphe neurons in *kiss1* mutants and siblings. ***A***, Change in fluorescence of GCaMP6f in neurons in the superior raphe of *kiss1*^sq1sj^ mutants (blue trace) and wild-type siblings (red trace). 50 V was applied for 1 s, 15 s after the start of imaging, at the point indicated by the arrow. The traces indicate mean value while the shading indicates SEM. ***B***, Average *z*-score of cells in the 15-s period after onset of shock. Each circle represents a single cell, and cells are arranged according to fish. The black dot indicates mean, and the bars indicate 95% confidence interval.

Kiss1 can increase the number of *c-fos*–expressing cells in the raphe (Ogawa et al., 2012), and the ability of Kiss1 to block response to the alarm substance is mediated by the raphe (Nathan et al., 2015b), which is downstream of the vHb. We thus asked whether loss of Kiss1 would affect activation of the raphe neurons. To find out, we performed calcium imaging and measured the response of raphe neurons to shock (Fig. 4). Given the nested design of the experiment (262 cells in 7 mutants and 221 cells in 6 wild-type fish), it is possible that differences may be due to genotype as well as an unknown factor (Aarts et al., 2014). To assess this, we determined the intracluster correlation. The value obtained was 0.456. Multilevel analysis was thus conducted, and this indicated that there is a large effect of genotype (Cohen’s *d* = 1.676). The 95% confidence interval of the effect of genotype was 0.241–1.434, i.e., higher than 0, indicating that the effect is significant. Calcium imaging thus suggests that loss of Kiss1 reduces activation of raphe neurons by an aversive stimulus.

## Discussion

In this article, we have tested the possibility that the neuromodulator Kisspeptin1 could participate in active avoidance learning in zebrafish. Kisspeptin1 is expressed only in the habenula of zebrafish (Kitahashi et al., 2009), primarily in ventral habenula neurons (Servili et al., 2011). The Kisspeptin1 receptor Kiss1ra (Onuma and Duan, 2012) is expressed in habenula neurons (Servili et al., 2011). The reduced ability of *kiss1* mutants to learn suggests that instrumental learning could involve signaling within the habenula. Given that loss of Kiss1 did not eliminate learning, other mechanisms must also be involved. These may include a change in input from the basal ganglia (entopeduncular nucleus) via feedback from the raphe (Amo et al., 2014), as well as other circuits such as those in the dorsal pallium (Aoki et al., 2013).

A function for Kisspeptin1 in regulating fear responses was suggested previously based on the finding that injection of the peptide into the brain ventricle of adult zebrafish led to depolarization of habenula neurons, as assessed by *c-fos* expression, and reduced expression of innate fear (Ogawa et al., 2014). Surprisingly, destruction of cells containing the Kisspeptin1 receptor, including vHb neurons, had the same behavioral effect as administering Kisspeptin1. This raises a conundrum: how can stimulating a neuron have the same effect as killing the neuron? The results here provide one way to resolve this puzzle, which is that Kisspeptin1 has a concentration-dependent effect on ventral habenula neurons: low concentrations lead to depolarization, and high concentrations lead to hyperpolarization. In principle, concentration-dependent modulation of neuronal membrane potential may result from the presence of different receptors with different affinities for the ligand, as has been shown for dopamine (Clemens et al., 2012). Alternatively, this effect may be achieved via a single receptor coupled to different downstream activators with distinct activation affinities.

Although the electrophysiological recordings reported here were performed at the larval stage, which provides optimal conditions for identifying and recording vHb neurons, this property of Kisspeptin1 signaling does not appear to be restricted to larval zebrafish. Ogawa et al. (2012) reported that 10^−11^ mol/g body weight of Kisspeptin1 increased *c-fos* expression in the habenula and raphe of adult zebrafish, whereas a higher concentration of 10^−9^ mol/g did not. Also, a concentration-dependent effect for Kisspeptin was reported in GnRH neurons of the medaka (Zhao and Wayne, 2012), with only low concentrations causing depolarization.

The experiments here demonstrate that Kisspeptin1 has the necessary properties to be involved in active avoidance by affecting vHb neurons. First, loss of the *kiss1* gene affects avoidance learning. Second, Kisspeptin1 has physiologic effects on vHb neurons. Third, loss of *kiss1* reduces activation of the raphe, which is downstream of the vHb. Successful learning may be accompanied first by Kisspeptin1-mediated excitation, and then by inhibition of habenula neurons. This would increase aversive expectation while the CS is being associated with US, and reduce aversive expectation once the strategy to avoid the US has been learned. What controls the release of Kisspeptin1 is unknown at present.

Aside from ventral habenula neurons, additional targets for Kisspeptin1 cannot be ruled out, as this modulator is expressed in habenula fibers that innervate the ventral interpeduncular nucleus (IPN; Servili et al., 2011). Kiss1rb, which has lower affinity for Kiss1 compared with Kiss1ra (Onuma and Duan, 2012), is expressed in the IPN (Servili et al., 2011). Distinguishing whether Kiss1 acts in the habenula or the IPN will require analysis of receptor mutants. At present, however, there is no evidence that the ventral IPN is required for avoidance learning; instead, the dorsal and intermediate IPN appear to be involved (Agetsuma et al., 2010). All evidence so far is thus consistent with the hypothesis that Kisspeptin1 functions as an intrinsic neuromodulator (Katz et al., 1994) of vHb neurons during avoidance learning.

## Synthesis

Reviewing Editor: Thomas McHugh, RIKEN Brain Science Institute Decisions are customarily a result of the Reviewing Editor and the peer reviewers coming together and discussing their recommendations until a consensus is reached. When revisions are invited, a fact-based synthesis statement explaining their decision and outlining what is needed to prepare a revision will be listed below. The following reviewer(s) agreed to reveal their identity: Satoshi Ogawa, Hidenori Aizawa.

Suppression of activity in the zebrafish ventral habenula, a structure homologous to the mammalian lateral habenula, reduces the avoidance ratio in an active avoidance task. Kisspeptin1 is a neuromodulator expressed in this structure. Previous work has shown that while central administration of kisspeptin1 suppressed the alarm substance-evoked fear response, inactivation of kisspeptin1 neurons also reduces fear response. Thus, while these data suggest a role of this neuropeptide in regulation of fish behaviors under the stress, contradictions remain.

In this study the authors focus on a role of kisspeptin1 in modulation of the ventral habenular neurons in zebrafish. They employ CRISPR/Cas9 technology to generate kisspeptin1 knockout alleles. Adult knockout fish fail to avoid shock that is predicted by light in active avoidance paradigm. This changes in behavior may be accounted for by the effect of kisspeptin1 on cellular response in the ventral hebenula, since the authors observed the ventral habenular neurons depolarized and hyperpolarized by low and high concentrations of kisspeptin1 in larva.

Although these data address an interesting cellular mechanism underlying a role of kisspeptin1 in regulation of fish behaviors, the manuscript still has multiple points which need further improvement to support its conclusions.

Main points:

1) While there is a clear behavioral difference between the mutant and control fish, it is still unclear whether this reduction is purely due to the reduction of learning ability. The authors fail to address other possibilities, such as a reduction of sensitivity of mutants to aversive stimuli. This should be addressed in control experiments. Further, it would be interesting to address the magnitude of the learning deficit by attempting additional training protocols. Specifically, with additional training will the mutants acquire the task?

2) The methods for the behavior should be clarified. What was the interval given between each active avoidance test? For effective learning, an intertrial interval may play a critical role. Does this behavioural paradigm contains intertrial interval?

3) The authors examined the behaviors using adult fish, while electrophysiology was done in larval fish. The authors should justify the use of different stage of the same lines for the two experiments.

4) It would be more straightforward to examine the changes in I-V relationship in electrophysiology of the ventral habenular neurons of kisspeptin1 mutant instead of pharmacological experiments done in Figure 3. This would provide a more direct link between the genetic manipulation and the impairment in avoidance behaviors.

5) In Figure 3, the authors showed the effect of K-10 and K-234 on the current response for a series of voltage changes in membrane potential using only 3-4 cells without statistical analysis. It is suggested to conclude based on the number of samples large enough for the statistical significance. This must be reasonable taking the possible heterogeneity of the lateral habenular neurons in mammals into consideration. Further, the details of kisspeptin1 (K-10) and kisspeptin receptor antagonist (K-234) used for the electrophysiology assay should be clarified. Is K-10 a synthesized peptide based on zebrafish kisspeptin1 sequence or mammalian (human or rodent) sequence? Agonistic effect of K-234 in zebrafish Kissrb has previously been shown? As far as I know, so far only one paper reported the antagonistic effects of synthetic kisspeptin analogs in a teleost fish, striped bass (https://www.ncbi.nlm.nih.gov/pubmed/26246220). As this study only demonstrated blockade effect of K-234 with one dose (1 uM), it remains unclear if K-234 really works as an antagonist for zebrafish Kiss-Rb.

6) It is still possible for the other brain regions than the ventral habenula to modulate avoidance behaviors. This possibility should be considered and discussed. For example, the ventral interpeduncular region expressing kisspeptin1 (Ogawa et al., 2014) is reported to be involved with fear behavior (Agetsuma et al., 2010).

7) It remains unclear how the kisspeptin1 act on activity of the neural circuit for avoidance behavior, since the authors did not examine the activity changes in the other brain regions connected with ventral habenula. In particular, activation of raphe nuclei in kisspeptin1 KO fish is of interest, since it receives direct inputs from the ventral habenula and send diverse outputs to the multiple brain regions. Checking them with c-fos expression may help.

Minor points

1) Description of the sex of the fish they used in the behaviors should be addeed.

2) For the electrophysiology assay, it is unclear how to confirm the successful targeting of kisspeptin neurons by patch clamp pipette. Any parameters applied for this?

3) The readers may not understand the mechanism underlying the effect of kisspeptin1 on the changes in membrane potential shown in Figure 3. Possible mechanism should be presented at least in Discussion section.
